# Production of Novel Bigels from Cold Pressed Chia Seed Oil By-Product: Application in Low-Fat Mayonnaise

**DOI:** 10.3390/foods13040574

**Published:** 2024-02-14

**Authors:** Abdulaziz S. Alkabaa, Alican Akcicek, Osman Taylan, Mohammed Balubaid, Mohammed Alamoudi, Waqar Ahmad Gulzar, Hisham Alidrisi, Enes Dertli, Salih Karasu

**Affiliations:** 1 Department of Industrial Engineering, Faculty of Engineering, King Abdulaziz University, Jeddah 21589, Saudi Arabia; aalkabaa@kau.edu.sa (A.S.A.); otaylan@kau.edu.sa (O.T.); mbalubaid@kau.edu.sa (M.B.); hmalidrisi@kau.edu.sa (H.A.); 2 Faculty of Tourism, Department of Gastronomy and Culinary Arts, Kocaeli University, Kartepe 41080, Turkey; a.akcicek@kocaeli.edu.tr; 3 Department of Food Engineering, Faculty of Chemical and Metallurgical Engineering, Yildiz Technical University, Davutpasa Campüs, Istanbul 34210, Turkey; edertli@yildiz.edu.tr

**Keywords:** Bigels, rheological properties, oloegel, hydrogel, chia seed gum, lowfat mayonnaise

## Abstract

The objective of this study was to produce an innovative bigel formulation by combining glycerol monostearate (GMS) oleogel with hydrogels stabilized by various agents, including cold pressed chia seed oil by-product gum (CSG), gelatin (G), and whey protein concentrate (WPC). The findings indicated that the choice of hydrogel influenced the rheological, textural, and microstructural properties of the bigels. The *G*′ value of the bigel samples was higher than *G*″, indicating that all the bigels exhibited solid-like characteristics. In order to numerically compare the dynamic rheological properties of the samples, *K*′ and *K*″ values were calculated using the power law model. *K*′ values of the samples were found to be higher than *K*″ values. The *K*′ value of bigel samples was significantly affected by the hydrogel (HG)/oleogel ratio (OG) and the type of stabilizing agent used in the hydrogel formulation. As the OG ratio of bigel samples increased, the *K*′ value increased significantly (*p* < 0.05). The texture values of the samples were significantly affected by the HG/OG ratio (*p* < 0.05). The study’s findings demonstrated that utilizing CSG, G, and WPC at an OG ratio more than 50% can result in bigels with the appropriate hardness and solid character. The low-fat mayonnaise was produced by using these bigels. The low-fat mayonnaise showed shear-thinning and solid-like behavior with *G*′ values greater than the *G*″ values. Low-fat mayonnaise produced with CSG bigels (CSGBs) showed similar rheological properties to the full-fat mayonnaise. The results showed that CSG could be used in a bigel formulation as a plant-based gum and CSGB could be used as a fat replacer in low-fat mayonnaise formulation.

## 1. Introduction

Emulsions are a highly popular category of food consumed by a wide range of individuals [1]. Food products, in particular emulsions, contain substantial quantities of saturated fat. Elevated intake of saturated fat is linked to adverse health outcomes including cardiovascular disorders, obesity, and elevated levels of LDL and cholesterol [2]. Therefore, it is advisable to decrease the amount of fat in food formulas or substitute saturated fat with alternative unsaturated fat sources [3]. Saturated fats have the greatest impact on the rheological, textural, sensory, and microstructural characteristics of emulsion-type foods [4]. The quality criteria are negatively affected by the decrease in fat content. Hence, there is a requirement for novel strategies that can substitute the attributes offered by saturated fat [5]. The food industry currently faces a significant challenge in the development of fat substitutes. Research on strategies to reduce saturated fat is progressing rapidly.

Biphasic gelation has recently emerged as a viable substitute for saturated fats, serving as a novel approach to structuring fats [6,7]. Biphasic gels, also known as bigels, are created by combining oleogelation and hydrogelation techniques [8]. This allows for the containment of both oil and water phases within a three-dimensional network [7]. Bigels offer enhanced physical stability compared to emulsions and emulsion gels by limiting the movement of the dispersed phase within the system. In addition, bigels possess a significant advantage in delivering both lipophilic and hydrophilic bioactive components due to their structure, which includes both an oil phase and a water phase [8,9,10,11]. However, the successful production of bigel with the desired rheological and textural qualities relies on numerous factors. The primary factors in the production of bigels are the specific type and ratio of gelling agents, as well as the ratio between hydrogel and oleogel [12]. The selecting the appropriate gelling agent is crucial for achieving the desired gel structure, particularly in the production of hydrogels [8]. Due to its ability to create hydrogen bonds between water, gelators, and non-gelling substances, gelatin is one of the most often employed proteins as hydrogelators in bigels [13,14]. Nevertheless, the high cost and ethical concerns associated with animal-derived resources necessitate the exploration of plant and less expensive alternatives [15].

Cold pressed oils contain high levels of bioactive compounds due to the absence of heat treatment or chemical refining processes during their production [16,17]. In recent years, there has been a growing tendency towards consuming foods that are not subjected to heat or chemical treatment, or have been subjected to minimal treatment [18,19]. Chia seed oil obtained through cold pressing is a rare plant resource abundant in omega-3 fatty acids. Therefore, the production of cold pressed chia seed oil has been exhibiting a notable rise in recent years [20]. Upon consumption of chia seed oil, its abundant protein and fiber content, as well as its cost-effective by-product, become evident [21,22,23]. Ongoing research indicates that chia seed gum has potential applications in food formulations [24,25]. Research has demonstrated that the polysaccharides present in the by-product of cold pressed chia seed oil possess the capacity to serve as an inexpensive gelling agent [21,23,26]. 

In the literature, there are many studies about bigels used in food application as animal-derived or dairy-based bigels [27,28,29,30,31,32,33]. However, the animal-origin proteins used in the production of bigels are not suitable for plant-based ingredients. [34]. Although numerous studies have been conducted in this field, there is still a need for further investigation into the plant-based bigels with a hydrogel: oleogel (HG: OG) ratio, in conjunction with the impact of gelator concentrations, particularly in the context of their potential application in food products. Considering this, limited studies were found which are about plant-based bigel production (HG: OG) as an alternative to dairy and animal-derived based bigels.

This study aimed to explore the feasibility of utilizing gum derived from the by-product of cold pressed chia seed oil as a substitute for gelatin and whey protein in the production of bigels. The by-product of cold pressed chia seed oil was utilized to extract gum, which was subsequently employed in the production of bigels. The rheological, texture, and microstructural properties of CSG-based bigels were compared to those of WPC and gelatin-based bigels. Therefore, the potential of CSG-based bigels as a gelling agent in bigel production was investigated. The second stage of this study focused on examining the capacity of reducing fat content in the production of low-fat mayonnaise using CSG-based bigels.

## 2. Materials and Methods

### 2.1. Materials

The glycerol monostearate, Whey Protein Concentrate, and gelatin (260–280 Bloom) were purchased from BADE Chemical (Izmir, Turkey), Slava Dairy (Karaman, Turkey) and Dairy Products and Gerede Jelatin (Bolu, Turkey), respectively. The refined blend of coconut oil was purchased from Oilnco Industries. The double distilled water was used for the analysis. All other chemicals were of analytical grade. CSOB was obtained from ONEVA Food Co. (Istanbul, Turkey). CSOB was derived by a mechanical screw press procedure. The chia seed was ground into flakes and dehydrated to a moisture content of 8%. Finally, a screw press was utilized to extract oil from chia seeds and acquire CSOB.

### 2.2. Methods

#### 2.2.1. Chia Seed Gum Extraction from Chia Seed Oil By-Product

The extraction of gum from cold pressed chia seed oil by-products followed the procedure outlined by Hijazi, Karasu [26]. First, 500 g of the by-products was mixed with 10 L of distilled water and heated to 80 °C for 2 h to form gel-like structures. The resulting gum solution was then filtered and purified by mixing it with three volumes of 96% ethanol. The gum was left to dry in an oven at 30 °C for 24 h. Once dry, the seed gums were packaged and stored under dry conditions at 4 °C.

#### 2.2.2. Preparation of Bigels

**Figure 1:**
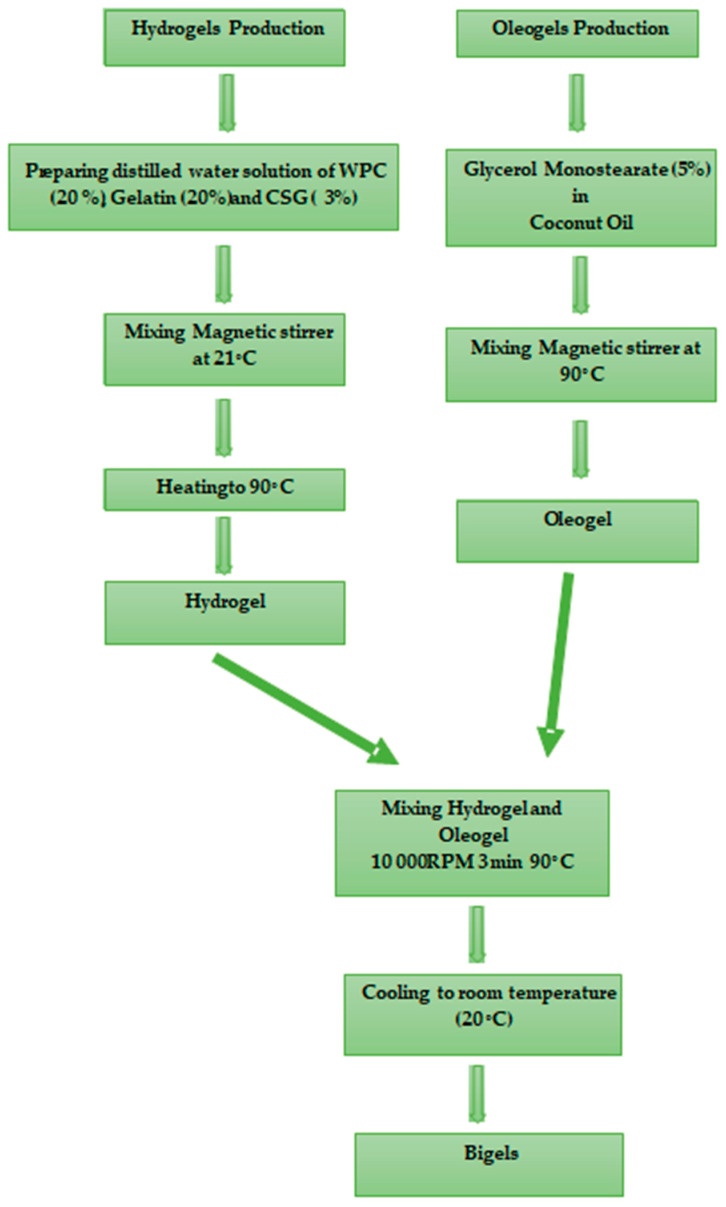
Hydrogel, oleogel, and bigel production stages.

The production of the bigels was carried out according to the method described by Martins, Guimarães [35]. The bigel production is illustrated in Figure 1. The hydrogel and oleogel phases of the bigels were separately prepared under different environmental conditions before being combined In the initial step, hydrogel samples were prepared by dispersing gelling agents (CSG, whey protein concentrate (WPC35), and gelatin) in distilled water at room temperature (~21 °C) using a mechanical mixer. The solutions were heated to approximately 90 ± 2 °C in a shaking water bath and maintained at 90 ± 2 °C for 1 h. Following that, the oleogel sample was produced. Initially, a 5% concentration of GMS was added to the refined coconut oil at 90° C and mixed at 300 rpm for 30 min [36]. GMS concentrations equal to or exceeding 5% by weight were necessary to ensure the minimum gelling concentration [35,37]. After oleogel production, the mixing process of hydrogel and oleogel phases was carried out using an Ultra Turrax homogenizer (Daihan, HG15 D, Wonju, Republic of Korea) for 3 min at 10,000 rpm to produce bigel samples. Following this period, the bigel samples were allowed to cool to room temperature and were subsequently stored at approximately 4 °C to initiate the structuring of the oil and water phases before undergoing testing. The compositions of various bigels and their corresponding names are presented in Table 1.

#### 2.2.3. Preparation of the Low-Fat and Full-Fat Mayonnaise

The low-fat and full-fat mayonnaise were prepared by using the modified method of Yang, Li [38]. To create low-fat mayonnaise, 30 g of the bigel samples was added to the deionized water. Then, 1 g sugar, 1 g salt, 5 g egg yolk powder, and 4 mL vinegar were dispersed in the aqueous phase. Then, 15 mL of sunflower oil was added to the aqueous solution. The mixture was then homogenized by using Ultra Turrax at 10,000 rpm for 1 min. The volume of the final mixture was 50 mL using deionized water. Meanwhile, a full-fat mayonnaise product serving as a control was prepared in a similar manner, consisting of 5 g of egg yolk, 65% rapeseed oil, 4 mL of vinegar, 1.0 g of salt, and 1.0 g of sugar [38,39]. Sodium benzoate was added as a preservative.

#### 2.2.4. Rheological Analyses

The rheological properties of low-fat and full-fat mayonnaise samples were investigated using a stress-controlled rheometer (MCR 302, Anton Paar, North Ryde, Austria) featuring a parallel-plate setup for shearing and a Peltier heating/cooling system. During these measurements, a PP50 probe with a 50 mm diameter was used (Anton Paar, North Ryde, Australia), and the gap between the plates was 0.7 mm for frequency sweep analysis and 0.5 mm for steady shear and 3-ITT analysis. Each rheological measurement was repeated three times at 25 °C.

This study encompassed an examination of steady shear rheological characteristics, ranging from shear rates of 0.1 s^−1^ to 100 s^−1^. The obtained data were modeled using the power law equation and fitted through nonlinear regression (Equation (1))

*τ* = *K* × γ^𝑛^ (1)

to determine parameters such as shear stress (*τ*) in Pa, consistency coefficient (*K*) in Pas^n^, shear rate (γ) in s^−1^, and flow behavior index (*n*).

The dynamic rheological properties of the oleogel, hydrogels, bigels, and mayonnaise were determined by using the stress-controlled rheometer with a Peltier heating/cooling system (MCR 302, Anton Paar, North Ryde, Austrialia). During these measurements, a PP50 probe with a 50 mm diameter was used (Anton Paar, North Ryde, Australia), and the gap between the plates was 0.7 mm for frequency sweep analysis and 0.5 mm for steady shear and 3-ITT analysis. Each rheological measurement was repeated three times at 25 °C.

The dynamic rheology of oleogels, hydrogels, and bigels was studied using a parallel-plate system. An amplitude sweep test was conducted at strain levels of 0.1% and 100% to identify the linear viscoelastic region. Following this, a frequency sweep test was performed within a frequency range of 0.1 to 10 Hz and an angular velocity range of 0.1 to 64 s^−1^. The storage modulus (*G*′) and loss modulus (*G*″) of the samples were determined based on their angular velocity. The dynamic rheological parameters were obtained by using the power law model and nonlinear regression analysis (Equations (2) and (3)) [40].

*G*′ = *K*′ × (ω)′ (2)

*G*″ = *K*″ × (ω)″ (3)

*G*′ represents the storage modulus (measured in Pa), *G*″ represents the loss modulus (measured in Pa), ω is angular velocity (measured in rad/s), and *K*′ and *K*″ denote the consistency coefficient values (expressed in Pas^n^), while n′ and n″ represent the flow behavior index values.

To investigate time-dependent thixotropic behavior under variable shear rates, a 3-interval Thixotropy Test (3-ITT) was employed, with shear rates of 0.5 s^−1^ and 150 s^−1^. The linear viscoelastic region was identified, ending at 50 s^−1^. The test involved applying a low shear rate (0.5 s^−1^) for the first 100 s, followed by an increase to 150 s^−1^ for 40 s in the second interval. The third interval focused on the dynamic rheological behavior of the mayonnaise during the second period. To model 3-ITT behavior, a second-order structural kinetic model was utilized, expressed by Equation (4).

(4) $$[\frac{G^{\prime }-G_{e}}{G_{0}-G_{e}}{]}^{1-n}=(n-1)k\times t-1$$

where *G*′ represents the change in the storage modulus, *G*_0_ is the initial storage modulus, *G*_e_ is the equilibrium storage modulus, *k* is the thixotropic velocity constant, and *t* is time.

#### 2.2.5. Textural Properties of Bigels

The textural characteristics of the oleogel, hydrogels, and bigel samples, which included measurements of hardness, springiness, and cohesiveness, were assessed using a Texture Analyzer (TA.XT2 Plus, Godalming, UK) equipped with a 50 kg load cell, and using a needle probe (5 mm in diameter). The process of preparing the bigels involved placing the hot gel into Petri dishes for the texture profile analysis. The penetration process involved the probe approaching the sample at a pre-test speed of 3.0 mm/s, transitioning to a test speed of 1.0 mm/s, with a trigger force set at 0.049 N. The probe penetrated the sample to a depth of 50% strain and subsequently returned to its initial position at a post-test speed of 3.0 mm/s. 

#### 2.2.6. Microstructural Properties

The morphology of the bigels was examined using a light microscope with 10× magnification (Olympus BX41, Tokyo, Japan). The process involved placing a coverslip over a small drop of the hot melting bigel sample on a microscope glass slide. After being stored at 4 °C for 24 h, the sample was subsequently observed under the microscope.

#### 2.2.7. Statistical Analysis

Each dataset underwent three parallel measurements, and every sample was produced in duplicate. Mean values were then calculated, and variations were assessed through standard deviations. For statistical analysis, the results were evaluated by utilizing the STATISTICA software program version 8 (Stat Soft Inc. in Tulsa, UK). Statistical evaluations were conducted using Duncan’s test to compare factor means, with a confidence level set at 95%. Additionally, rheological properties involved the application of nonlinear regression analysis. Nonlinear regression analysis was applied to determine the parameters of the power law model using STATISTICA software (Stat Soft Inc. in Tulsa, UK).

## 3. Result and Discussion

### 3.1. Dynamic Rheological Properties of Oleogels and Hydrogels

**Figure 2:**
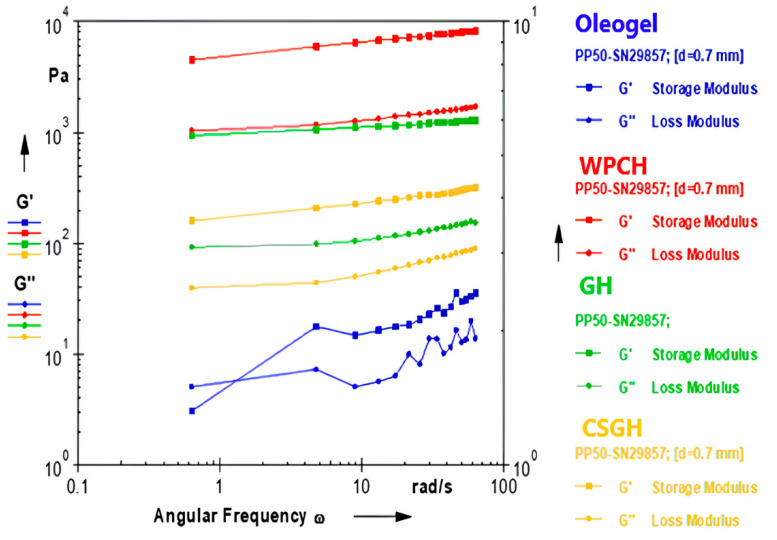
Dynamic rheological properties of oleogel and hydrogel samples (CSGH: hydrogel produced from cold pressed chia seed oil by-product gum, GH: Gelatin Hydrogel, WPCH: Whey Protein Concentrate Hydrogel).

Figure 2 displays the dynamic rheological properties of the hydrogel and oleogel samples. The *G*′ value surpasses the *G*″ value over the whole frequency range for all hydrogel samples. The findings indicate that all of the hydrogel samples demonstrated a solid-like structure, which is expected from hydrogel materials. There was no significant rise in the *G*′ value of the all hydrogels with increasing frequency level. The results demonstrate that the hydrogel structures created possess significant strength. Alves Barroso, Grossi Bovi Karatay [41] characterized the rheological properties of potato starch bigels. The researchers found that the *G*′ value of the hydrogel samples exceeded the *G*″ value across the whole frequency range. In addition, they highlighted that the *G*′ value of bigel samples remains constant regardless of frequency, indicating that there is no considerable variation in *G*′ with an increase in frequency. They reported that the solid nature of the bigel samples was predominant, similar to our results. Zheng, Mao [42] reported that the storage modulus (*G*′) of the bio gels (BGs) containing GMS and κ-carrageen was much higher than the loss modulus (*G*″), suggesting the formation of elastic gel networks. The oleogel sample demonstrates that the *G*′ value surpasses the *G*″ value throughout all frequency ranges, except the initial frequency. An elevated rate of rise in the *G*′ and *G*″ values was seen when the frequency increased in the oleogel samples, in contrast to the hydrogel samples. However, this upward trend is a phenomenon that may be readily recognized in gel samples [43]. The frequency dependence of the oleogel sample suggests that its gel network is comparatively weaker than that of all hydrogel samples, including CSGH. The WPCH exhibited the highest *G*′ value when compared to other hydrogels. CSGH exhibited the lowest *G*′ value in the whole frequency range. However, the CSGH consistently displayed a greater *G*′ value compared to the oleogel sample across all frequencies, indicating its suitability for bigel production. G and WPC were utilized at a proportion of 20% in the production of hydrogels, while CSG was employed at only 3%. The result suggests that CSG is capable of achieving the required hydrogel structure at low concentrations, which can be regarded as a significant property for gelling agents [44,45]. 

The dynamic rheological characteristics of hydrogel samples were quantitatively interpreted by modeling the frequency sweep data using a power law model. The parameters of the power law model are presented in Table 2. The experimental results revealed that the *K*′ value, which represents the measure of firmness, exhibited greater values compared to the *K*″ value, which represents the measure of liquid structure, across all hydrogel samples. Consequently, it was determined that the hydrogels created exhibited the desired solid properties. The *K*′ values of all hydrogel samples were found to be higher than the *K*′ value of the oleogel sample. However, the fact that the *K*′ value of all gel samples, including oleogel, is much higher than *K*″ shows that the solid character of all the gel samples, which is desired from the gel structure, is more dominant than the liquid characters [46]. When we compared the hydrogel samples, WPCH had the highest *K*′ value, while CSGH had the lowest *K*′ value. These findings are consistent with the *G*′ data.

### 3.2. Dynamic Rheological Properties of Oleogels, Hydrogels, and Bigels

**Figure 3:**
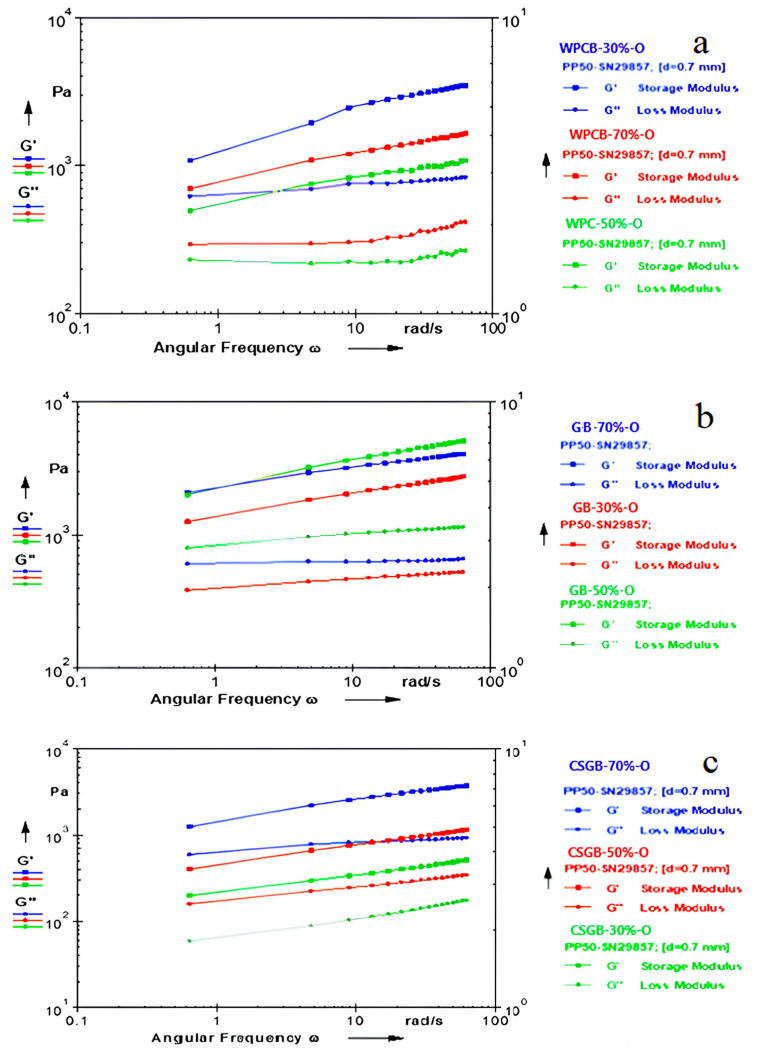
Dynamic rheological properties of the bigel samples ((**c**) CSGB-70%-O, CSGB -50%-O, CSGB-30%-O: samples produced with cold pressed chia seed oil by-product gum at different oleogel concentrations, (**b**) GB-70%-O, GB-50%-O, GB-30%-O: samples contained gelatin at different oleogel concentrations, (**a**) WPCB-70%-O, WPCB-50%-O, WPCB-30%-O: samples contained Whey Protein Concentrate at different oleogel concentrations. CSGH: hydrogel produced from cold pressed chia seed oil by-product gum, GH: Gelatin Hydrogel, WPCH: Whey Protein Concentrate Hydrogel).

The viscoelastic characteristics of bigel samples produced using WPC, CSG, and G are displayed in Figure 3. The *G*′ value exhibited much greater than the *G*″ value in all frequency levels for all bigel samples. The findings of this study indicate that all samples of bigel had viscoelastic solid characteristics, irrespective of the HG-OG ratio. In addition, the *G*′ and *G*″ values of the bigel samples were considerably affected by the HG-OG ratio. With an increase in the OG ratio, there was a considerable increase in the *G*′ value, indicating that an increase in the OG ratio led to an increase in the solid-like behavior. Fernandes, Neves [47] reported that bigels showed solid-like behavior regardless of the HG-OG ratio. In their study, a more solid-like structure was obtained at higher OG ratios. Zheng, Mao [42] reported that bigels obtained from potato starch showed solid-like behavior in a similar manner to our study. It was also reported that the samples with the greatest hydrogel ratio had the lowest *G*′ value among all the bigel samples. Patel, Mankoč [48] reported the magnitude of *G*′ increased notably in bigel systems with elevated oleogel fractions. 

Figure 3 demonstrates that the HG/OG ratio and the selection of gelling agent in hydrogel production strongly influenced the gel structures of the bigel samples. The bigel samples produced with WPC exhibited the greatest *G*′ value when the HG-OG ratio was 50%. The *G*′ values in all bigel samples made with WPC exhibited a noticeable rise as the frequency increased. For the samples produced with G and CSG, the *G*′ value exhibited a rather slight rise as the frequency rose. The findings of this study indicate that the viscoelastic characteristics of bigel samples made with WPC are comparatively weaker when compared to CSGB and GB samples, which displayed a similar trend in frequency. The sample that exhibited the greatest *G*′ value was derived from the hydrogel–oleogel mixture with a ratio of 50%–50%. Nevertheless, the *G*′ value exhibited a higher degree of dependence on the frequency value within the present sample, with a range of 50% for HG and 50% for OG. In the sample including a mixture of 30–70% HG/OG, the storage modulus (*G*′) exhibited a value that is similar to that of a sample consisting of an equal proportion (50/50%) of HG and OG. The sample with a composition of 30–70% HG/OG exhibited a much lower level of dependence on the increase in frequency. Upon evaluating these two criteria, it can be concluded that the sample with a 30–70% HG/OG composition has a greater degree of elasticity. A higher OG ratio results in the development of a gel structure characterized by enhanced elasticity. The rheological properties of the bigels appeared to be mainly affected by the oleogel component, potentially leading to higher gel strength in comparison to the individual oleogels [47,48]. 

The sample produced by CSG exhibited the maximum *G*′ value at a higher OG ratio. In samples of CSGB with a high OG ratio, the *G*′ value exhibited a lower degree of frequency-dependent variation. Therefore, the oleogel ratio emerged as the primary factor influencing the development of gel strength in the samples which were made using CSG and G. This finding is consistent with other research conducted using the bigel system. Zheng, Mao [42] and Rehman, Amin [49] reported that the *G*′ values of the bigels exhibited a slight rise as the frequency level increased, and this increase was more pronounced in bigel systems with greater proportions of hydrogels. This observation implies that bigels containing a higher fraction of hydrogels tend to have increased viscosity and reduced elasticity compared to those with lower hydrogel content. The investigation yielded comparable findings. These findings indicate that when hydrogels and oleogels are mixed together in the bigels, they exhibit a synergistic effect. The observed phenomenon can be attributed to the combining and synergistic effect of the oleogel and hydrogel networks present in the bigels [42,48,50,51]. This phenomenon is likely due to the emulsifier molecules affecting how the oleogel and hydrogel interact with each other and how the oleogel forms. These interactions have an impact on the development of the three-dimensional structure within the bigel matrix [51]. 

The viscoelastic behavior of the bigel samples varied according to the type of gelling agent used in hydrogel production. CSGB revealed *G*′ values comparable to the GB and WPCB, while the hydrogel prepared with CSG exhibited lower *G*′ than the hydrogel samples prepared with G and WPC. This shows that in bigel production, CSGH can exhibit strong interaction with oleogels and provide the required gel network. The bigel samples that were produced with CSG and G demonstrated a greater prevalence of elastic characteristics, but possessed a lower hydrogel strength in comparison to the bigel samples prepared using WPC. The findings indicate the presence of a synergistic effect between the hydrogel and oleogel in GB and CSGB samples.

The frequency sweep test values of bigel samples were subjected to modeling using the power law model, resulting in the calculation of *K*′ and *K*″ values (Table 2). A greater value of *K*′ suggests a greater dominance of the elastic characteristic. Table 2 displays the observed *K*′ values, which exhibit significant variations among the different samples (*p* < 0.05). The *K*′ values of the all bigel samples were higher than the *K*″ values, indicating that all bigel samples showed viscoelastic solid character. The *K*′ values of the bigels produced with CSG, G, and WPC were found to be 227.14–1552.92 Pas^n^, 1632.11–2671 Pas^n^, and 542.51–1440.21 Pas^n^. Considering the maximum *K*′ value, GB showed the highest *K*′ values while WPCB exhibited the lowest *K*′ value. The sample produced with WPC had the highest *K*′ value with a HG/OG ratio of 50/50 while the sample prepared with G and CSG resulted in the highest *K*′ value with a HG/OG ratio of 30/70. In contrast to WPCB, the *K*′ values of all G and CSG bigels showed higher values than those of their hydrogels. In addition, CSGB exhibited higher *K*′ values than WPCB, unlike its hydrogel sample. This result indicated that CSG could show a more compact structure in bigel production compared to WPC. The frequency sweep test demonstrates that the CSGB samples exhibited the desired viscoelasticity and yielded comparable results to samples obtained with WPC and G. Therefore, it has been concluded that CSG can be cheaply employed in the manufacturing of bigels with the intended rheological characteristics.

### 3.3. Textural Properties of Bigels

The texture profile analysis (TPA) offered valuable data regarding characteristics such as hardness, cohesiveness, and springiness. Textural properties of the oleogel, hydrogels, and bigels are presented in Table 3. The textural properties were significantly influenced by the gelling type and the HG/OG ratio. 

The hardness values of the bigel samples ranged from 749.78 to 3849.62 g for CSGB, 1758.36 to 4300 g for GB, and 645 to 3330.94 g for WPCB. These values were greater than those of the oleogel sample. Furthermore, it was observed that the hardness value of all bigel samples, with the exception of the bigel samples made with 30% CSG and WPI, exhibited greater hardness compared to their respective hydrogels. Both the HG/OG ratio and the choice of gelling agent exhibited significant effects on the hardness values of bigel samples (*p* < 0.05). The hardness value of the bigels increased with an increase in the OG/HG ratio. The samples with an OG/HG ratio of 30% exhibited the lowest hardness value among all the bigel samples. A notable increase in the hardness value was seen across all samples of bigel as the OG ratio increased from 30% to 50%. However, this rise exhibited a lowering trend as the OG ratio further increased from 50% to 70%. The correlation between the increase in hardness value and the rise in OG ratio has been documented by other researchers [46,52,53]. The increase in the oil phase fraction resulted in the enlargement of the oil droplets and the enhancement of the structural organization within the systems. Consequently, the use of structured oleogels served as active fillers, enhancing the connectivity of the network and resulting in improved hardness throughout the matrix [42,46]. Li, Han [52] found that increasing OG fractions significantly improved the hardness value of the bigels. This implies the significant role played by the rigid oleogel structure within the bigel systems and suggests a synergistic effect in strengthening the bigel structures. 

CSGB samples showed a higher hardness value than WPCB samples and exhibited a comparable hardness value with GB samples, indicating that CSGB showed acceptable hardness characteristics. While initially having the lowest hardness value, the CSG hydrogel’s bigel samples exhibited a significant improvement in hardness to 3849.62 at a 70% OG/HG ratio. These findings indicate that CSG had a favorable interaction with oleogel during its production of bigel and resulted in a more compact structure. Springiness refers to how food can recover its original shape after being altered or deformed. The variation in springiness values among bigels with varying OG/HG ratios was minimal [51]. The springiness response of all the bigel samples consistently displayed values exceeding 90%. This indicates that the interactions between the oleogel and hydrogel, especially in the presence of G, WPC, and CSG, played a role in enhancing the strength of the gel system. These observations suggest that the dispersed phase in bigels acted as a reinforcing filler to increase their strength [51,54]. This underscores a significant trait, signifying the bigels’ ability to demonstrate remarkable resilience and recovery capacity. Similar results were reported by Martins, Guimarães [35]. The cohesiveness values of all samples showed significant differences due to the behavior being a consequence of modifications within the internal network resulting from the first deformation. Consequently, oleogel particles/droplets were not able to display higher mobility (due to increased gel viscosity), thus significantly affecting this parameter [35]. The single oleogel demonstrated higher hardness values compared to the single hydrogel. When compared to both the individual oleogel and hydrogel, the bigels displayed intermediate levels of hardness, springiness, and cohesiveness. Consequently, the CSGB samples exhibited the intended textural characteristics.

### 3.4. Microstructural Properties

**Figure 4:**
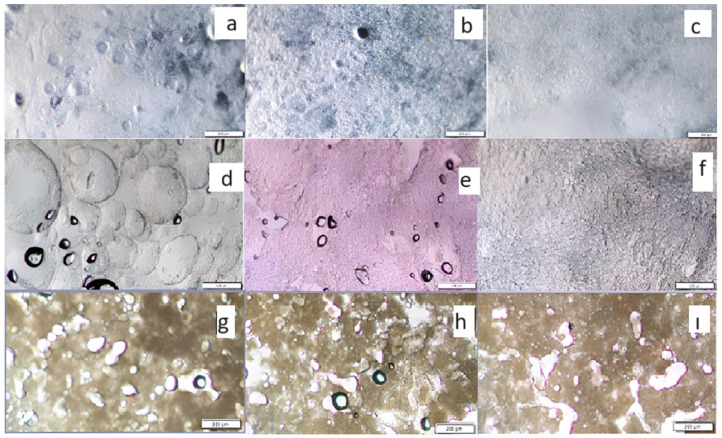
Optical microscope images (**a**) CSGB -70%-O, (**b**) CSGB -50%-O, (**c**) CSGB-30%-O: samples produced with cold pressed chia seed oil by-product gum at different oleogel concentrations, (**d**) GB-70%-O, (**e**) GB-50%-O, (**f**) GB-30%-O: samples contained gelatin at different oleogel concentrations, (**g**) WPCB-70%-O, (**h**) WPCB-50%-O, (**i**) WPCB-30%-O: samples contained Whey Protein Concentrate at different oleogel concentrations. CSGH: hydrogel produced from cold pressed chia seed oil by-product gum, GH: Gelatin Hydrogel, WPCH: Whey Protein Concentrate Hydrogel.

Optical microscopic images of bigels, characterized by different OG/HG ratios and emulsifiers types, are depicted in Figure 4. The microstructure of bigels varied by OG/HG ratios and gelling type. In addition, an increase in the OG ratio resulted in a corresponding increase in droplet size. This was substantiated by the visual evidence provided in the micrographs, which clearly depicted the presence of bigger droplets. Similar results were reported by previously published studies [42,53]. The The larger droplets can be explained by a higher OG ratio, which in turn increased the system’s viscoelastic properties [53]. Typically, the probability for coalescence events is higher in larger oleogel droplets, whereas the absence of structural uniformity in bigels might result in variances in mechanical characteristics. Therefore, when incorporated into a complex food structure, this component can significantly influence the overall stability and properties of the bigels. [35]. In addition, CSGB bigels showed higher stability with smooth surface than the GB and WPCB samples. However, increasing the oleogel ratio formed a more complex bigel structure for all bigel samples. As the proportion of oleogels reached 50 and 70 wt%, the droplets became densely packed, and a higher oleogel content caused the formation of larger droplets. This close packing phenomenon promoted the aggregation of droplets, resulting in a more pronounced variation and uneven distribution of particles [53].

Microscopic images of all these bigels revealed the presence of small emulsion-like droplets. These particles were mostly irregular in shape, and their size remained relatively consistent as the OG/HG ratios varied from 3:7 to 7:3. Similar results were reported by [51]. In addition to the observed circular or spherical particles, a central core composed of ellipse-like elements was generated during the process of physically trapping the oleogel (Figure 3). This phenomenon can be attributed to the elevated viscosity of the continuous (aqueous) medium [35].

### 3.5. Rheological Properties of Mayonnaise Samples Prepared from Bigels

#### 3.5.1. Steady Shear Rheological Properties

**Figure 5:**
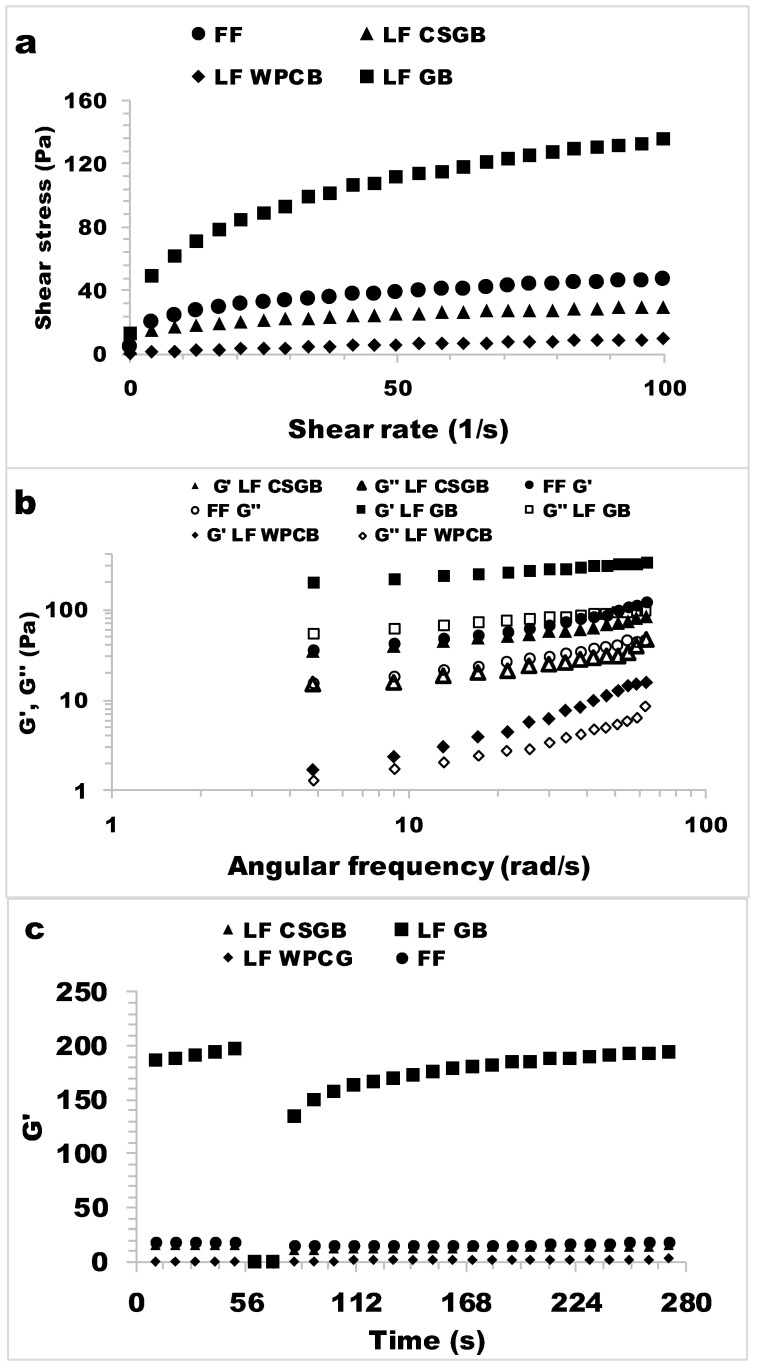
Steady shear (**a**), dynamic (**b**), and 3 ITT (**c**) rheological properties of low-fat mayonnaise produced from Bigel. (*FF: Full Fat mayonnaise with 65% Oil, LF CSGB: Low-fat mayonnaise samples contained CSGB and 30% Oil, LF GB: Low-fat mayonnaise samples contained GB and 30% Oil, LF WPCB: Low-fat mayonnaise samples contained Whey Protein Concentrate Bigel and 30% Oil)*.

The flow curves of the LF (low-fat) mayonnaise samples produced with different bigels and FF (full-fat) mayonnaise are given in Figure 5a. An increase in the shear rate caused a decrease in viscosity, which implied non-Newtonian shear-thinning behavior for the mayonnaise samples [38,55,56]. These phenomena may be explained by the applied force breaking weak bonds between molecules in the product, decreasing the connection between the components and enhancing the alignment of the constituent molecules [57]. 

The LF GB mayonnaise sample exhibited a higher shear rate, indicating a higher viscosity value, compared to the other mayonnaise samples within the whole shear rate range. The LF-GB samples had the highest level of pseudoplasticity among the others. The LF CSGB mayonnaise demonstrated a flow behavior profile that was comparable to that of FF mayonnaise, suggesting that CSGB might be employed as a substitute for fat in the production of low-fat mayonnaise. The LF WPCB mayonnaise samples exhibited less pronounced pseudoplastic behavior in comparison to the FF and LF CSGB samples. The power law model was used to model the steady shear rheological properties of mayonnaise samples. The rheological properties of all the mayonnaises were determined based on the well-fitted power law model parameters, which exhibited high regression coefficients (R^2^: 0.995–0.999). *K* values ranged from 0.75 to 21.71 Pas^n^, with the LF GB sample having the highest *K* value and the LF WPCB sample having the lowest (Table 4). However, the n values varied between 0.29 and 0.95. The n values variation indicates that all the samples exhibited a shear-thinning rheological behavior [58]. These findings indicate that all the tested mayonnaise samples exhibited pseudoplastic behavior (*n* < 1). A significant reduction in the proportion of oil within emulsions, as observed in products like mayonnaise and salad dressing, leads to a marked decrease in their overall thickness and consistency. It is crucial to compensate for this reduction, especially when formulating low-fat alternatives [58,59]. The lowest *K* value and the highest *n* value were observed for the LF WPC mayonnaise (0.75 Pas^n^). In addition, the *K* value of the LF GB was found to be higher than the FF mayonnaise and showed the highest *K* value among the mayonnaise samples. The significant rise in *K* value may be ascribed to the synergistic interaction between HG and OG in the formulation of mayonnaise. [42,51]. The LF GB mayonnaise sample and FF mayonnaise samples exhibited *K* values of 7.95 Pas^n^ and 9.11 Pas^n^, and n values of 0.29 and 0.32, respectively. There was no significant difference observed in the *K* and *n* values between the LF GB and FF mayonnaise samples (*p* < 0.05). A higher *K* value and a lower n value can be regarded as an indication of strong pseudoplastic character. However, the flow behavior exhibited by mayonnaise is not typically characterized by an extremely high *K* value, such as the one observed in the LF GB sample. Therefore, the primary factor to consider in this case should be the closeness in terms of the *K* value and *n* value of the full-fat sample (FF). The LF CSGB sample displayed *K* and n values that were similar to those of the FF sample, indicating that CSGBs could be successfully used in low-fat mayonnaise production as plant-based fat replacers. The flow behavior index value (*n*) being less than 1 indicates that all the model systems behave as non-Newtonian fluids, displaying pseudoplastic characteristics. Notably, the mayonnaises containing LF CSGB exhibited a more pronounced shear-thinning behavior, indicated by a smaller *n* value (0.29), compared to the regular FF (0.32) mayonnaises. This enhanced shear-thinning behavior may be attributed to the disruption of hydrocolloids caused by mechanical energy, followed by their reaggregation into smaller aggregates [55]. The LF WPCB mayonnaise sample displayed the highest *n* value (close to 1) value and an extremely low *K* value, indicating that with a weaker pseudoplastic character, WPCB is unsuitable as a fat-reducing agent in the preparation of mayonnaise. 

#### 3.5.2. Dynamic Rheological Properties

The frequency sweep test results for LF and FF mayonnaise samples are shown in Figure 5b. The *G*′ value for all mayonnaise samples exceeds the *G*″ value across the whole frequency range [58]. According to the findings, all mayonnaise samples had a solid-like structure. This is to be expected from LF and FF mayonnaise samples. The solid-like characteristic of the mayonnaise samples was also reported from other studies [55,58]. According to Figure 5b, the LF CSGB and LF GB seem to strengthen the gel structure of mayonnaise. Particularly, low-fat samples containing LF GB displayed significantly higher *G*′ values compared to the FF mayonnaise sample. Also, Figure 5b demonstrates that the mayonnaise sample made with WPCB has a lower *G*′ value than the other mayonnaise samples, while the mayonnaise sample made with GB has a greater *G*′ value. As the frequency increases, the increase in *G*′ values tends to decrease from the samples with the lowest *G*′ (WPCB) to those with the greatest *G*′ (G). LF CSGB and LF GB mayonnaise samples showed similar frequency dependence. Likewise, the relationship between *G*′ and *G*″ values in the bigels demonstrated a diminishing dependency on oscillatory frequency as the increased frequency is shown, suggesting an enhancement of the gel matrix for the LF GB and LF CSGB mayonnaise which is used in mayonnaise formulation [60]. However, *G*′ and *G*″ of the LF- CSGB mayonnaise were more similar to that of FF mayonnaise.

The findings demonstrate the substantial potential of the CSGB in a production of LF mayonnaises. A power law model was used to describe frequency sweep data to quantitatively assess the dynamic rheological characteristics of mayonnaise samples. Table 4 exhibited the parameters of the power law model. The samples displayed *K*′ and *K*″ values ranging from 0.166 to 145.60 and 0.083 to 39.42 Pas^n^, respectively, with n′ and n″ values in the range of 0.118–0.224 and 0.158–0.290 (R^2^ > 0.99). In all mayonnaise samples, *K*′ exceeded *K*″, indicating a dominance of elastic solid behavior over viscous characteristics [58]. The *K*′ value, which represents the measure of elasticity, is larger than the *K*″ value, which indicates the measure of the liquid structure, according to experimental results. Remarkably, LF CSGB and LF GB displayed higher *K*′ and *K*″ values than the full-fat control sample and significantly impacted the viscoelastic behavior of low-fat mayonnaise samples. When we compared the mayonnaise samples, we found that LF WPCB had the lowest *K*′ value (0.166 Pas^n^), while LF GB had the highest *K*′ value (145.60 Pas^n^). Also, LF CSGB showed the closest *K*′ (19.48 Pas^n^) values to FF mayonnaise (11.31 Pas^n^). However, *K*′ of the LF CSGB values showed significant differences and *K*″ showed no significant differences from the FF mayonnaise samples. This outcome is consistent with the *G*′ data. It was concluded as a result that the CSGB-based mayonnaise samples displayed the requisite solid characteristics.

#### 3.5.3. 3-ITT

The 3-ITT characteristics of the mayonnaise samples are given in Figure 5c, and show that the degree of recovery of the sample as a result of a sudden deformation varies depending on the applied shear rate, in other words, the deformation value. As seen in Figure 5c, a dramatic decrease in *G*′ value was observed in LF CSGB, LF GB, and FF mayonnaise samples with the effect of sudden deformation applied in seconds time intervals. In the third time interval, a different degree of recovery was observed in all products. The recovery trend after deformation is an important quality criterion for these products. However, LF CSGB showed similar recovery properties to FF mayonnaise. In addition, despite the decrease in recovery abilities expected with a reduction in oil content, a complete recovery was observed in low-fat mayonnaise samples stabilized with CSGB and GB. Similar results were found using the WPI-EPS complex for low-fat mayonnaises [58,61]. This result indicated that the samples stabilized with CSGB and GB could show a sufficient recovery ability as well as the desired consistency and viscosity.

Table 4 provides data on three key parameters derived from the second-order structural kinetic model: *G*_0_, *G_e_*, *G_e_*/*G*_0_, and *k* × 1000. The observed ranges for these parameters, as well as for R^2^, were as follows: *G*_0_ (16.52–135), *G_e_* (33.3–205), *G_e_*/*G*_0_ (1.51–2), *k* × 1000 (4.52–5.2), and R^2^ (>0.91–0.99). It is worth noting that both FF mayonnaise and LF CSGB mayonnaise showed similar thixotrophic behavior with no significant differences (*p* < 0.05), as evidenced by their higher values of *G_e_*/*G*_0_ and *k*. Also, it is worth noting LF CSGB mayonnaise showed a near-perfect thixotropic recovery to FF mayonnaise. The findings indicate that CSGB has the potential to enhance the thixotropic characteristics of low-fat mayonnaise. These findings revealed that LF CSGB mayonnaise samples may retain their viscoelastic properties after food preparation involving a considerable amount of sudden deformation, such as homogenization or pumping, as well as consumption while shaking and squeezing [26,62]. Similar results were reported for low-fat mayonnaise production [26,61].

Rheological tests indicate that LF CSGB and FF mayonnaise samples share comparable rheological characteristics, including similar flow behavior, viscoelastic behavior, and thixotropic properties. Similar results were reported by Yang, Gong [63] and Yang, Li [38] for the sodium alginate and konjac glucomannan emulsion gels, and egg yolk systems were used in the formulation of low-fat mayonnaise samples. These findings suggest that the combined LF CSGB systems exhibit favorable viscoelastic, thixotropic, and plastic properties, underscoring their promising potential for use in the production of bigels resembling low-fat mayonnaise.

## 4. Conclusions

During the process of producing cold pressed oil, by-products that include significant amounts of protein, fiber, and bioactive components are generated. The transformation of these by-products into high-value products, such as innovative additives, is highly important for both oil producers and other collaborators in the food sector. The gum utilized in this investigation was derived from the by-product of cold pressed chia seed oil extraction. The investigation focused on the utilization of the acquired gum as a stabilizing agent in the manufacturing of bigel. Furthermore, this study investigated the impact of including prepared bigel on the reduction in fat content during the manufacturing of low-fat mayonnaise. The rheological, textural, and microstructural characteristics of the bigel samples were influenced by both the OG/HG ratio and the type of stabilizing agent employed. Notably, an increase in the OG/HG ratio resulted in higher values for G′, *K*′, and hardness. The frequency sweep test revealed that the solid character of WPC hydrogel was higher than that of G and CSG hydrogel, while the *K*′ value of G and CSG bigels was higher than that of WPC bigels. These results concluded that, like GH, CSGH has the potential to be utilized in the manufacturing of bigel with a desired viscoelastic structure. The low-fat mayonnaise sample prepared with CSGB exhibited similar *K* (7.95 Pas^n^), *K*′ (19.48 Pas^n^), and *Ge*′/*Go*′ (1.97) values to the full control mayonnaise sample, which had *K* (9.11 Pas^n^), *K*′ (11.31 Pas^n^), and *Ge*′/*Go*′ (2.00). LF CSGB showed similar flow behavior, viscoelasticity, and recoverable character to FF mayonnaise. The results suggested the CSGB effectively compensated for the absence of fat in low-fat mayonnaise. The findings of this study indicated that CSG exhibits potential as a plant-derived resource for the production of bigels and CSGB produced with 50% and 70% OG could be utilized in the formulation of low-fat mayonnaise.

## Figures and Tables

**Table 1:** All formulations of oleogels, hydrogels, and bigels.

Sample Name	Emulsifier	Hydrogel:Oleogel Ratio
Oleogel	-	0:100
CSGB-70%-O	Chia Seed Gum (3%)	30:70
CSGB-50%-O	Chia Seed Gum (3%)	50:50
CSGB-30%-O	Chia Seed Gum (3%)	70:30
GB-70%-O	Gelatin	30:70
GB-50%-O	Gelatin	50:50
GB-30%-O	Gelatin	70:30
WPCB-70%-O	WPC35	30:70
WPCB-50%-O	WPC35	50:50
WPCB-30%-O	WPC35	70:30
CSGH	Chia Seed Gum (%3)	100:0
GH	Gelatin	100:0
WPCH	WPC35	100:0

(*Oleogel: samples contained 5% GMS, B-70%-O-CSG B-50%-O-CSG B-30%-O-CSG: samples contained chia seed gum at different oleogel concentrations, B-70%-O-G B-50%-O-G B-30%-O-G: samples contained gelatin at different oleogel concentrations, B-70%-O-WPC B-50%-O-WPC B-30%-O-WPC: samples contained Whey Protein Concentrate at different oleogel concentrations. Hydrogel-CSG: chia seed gum hydrogel, Hydrogel-G: Gelatin Hydrogel, Hydrogel-WPC: Whey Protein Concentrate Hydrogel*).

**Table 2:** The Power Law Parameters of Dynamic Rheological Parameters f of Oleogel, Hydrogel and Bigels.

Sample Name	*K*′ (Pas^n^)	n′	R^2^	*K*″ (Pas^n^)	n″	R^2^
Oleogel	5.333± 0.345 ^f^	0.444 ± 0.03	0.947	2.265 ± 0.136 ^g^	0.083 ± 0.01	0.932
CSGB-70%-O	1552.92 ± 24.61 ^c^	0.215 ± 0.01	0.997	660.29 ± 26.010 ^c^	0.313 ± 0.004	0.996
CSGB-50%-O	461.04 ± 7.81 ^def^	0.220 ± 0.013	0.999	198.60 ± 17.012 ^ef^	0.171 ± 0.012	0.997
CSGB-30%-O	227.14 ± 18.01 ^ef^	0.202 ± 0.03	0.998	60.46 ± 22.172 ^efg^	0.251 ± 0.028	0.996
GB-70%-O	2671.68 ± 490.991 ^b^	0.138 ± 0.005	0.996	730.35 ± 175.686 ^bc^	0.011 ± 0.006	0.854
GB-50%-O	2347.90 ± 348.62 ^b^	0.186 ± 0.005	0.997	850.74 ± 78.43 ^ab^	0.072 ± 0.005	0.991
GB -30%-O	1632.11 ± 317.244 ^c^	0.156 ± 0.004	0.999	456.48 ± 83.471 ^d^	0.06 ± 0.006	0.999
WPC B-70%-O	701.69 ± 142.719 ^de^	0.179 ± 0.007	0.995	221.89 ± 51.337 ^e^	0.115 ± 0.023	0.883
WPC B-50%-O	1440.21 ± 97.54 ^c^	0.218 ± 0.003	0.989	636.274 ± 68.45 ^c^	0.063 ± 0.005	0.987
WPC B-30%-O	542.51 ± 48.333 ^def^	0.149 ± 0.002	0.982	194.40 ± 20.633 ^ef^	0.049 ± 0.009	0.985
CSGH	140.94 ± 16.82 ^ef^	0.165 ± 0.101	0.998	25.09 ± 6.56 ^fg^	0.289 ± 0.01	0.988
GH	1111.09 ± 219.205 ^cd^	0.064 ± 0.006	0.979	83.88 ± 6.434 ^efg^	0.159 ± 0.010	0.946
WPCH	4829.41 ± 429.47 ^a^	0.129 ± 0.009	0.999	989.82 ± 57.39 ^a^	0.124 ± 0.008	0.982

a–g: Different superscript letters indicate significant differences between samples (*p* < 0.05). (CSGB-70%-O, CSGB-50%-O, CSGB-30%-O: samples produced with cold pressed chia seed oil by-product gum at different oleogel concentrations, GB-70%-O, GB-50%-O, GB-30%-O: samples contained gelatin at different oleogel concentrations, WPCB-70%-O, WPCB-50%-O, WPCB-30%-O: samples contained Whey Protein Concentrate at different oleogel concentrations. CSGH: hydrogel produced from cold pressed chia seed oil by-product gum, GH: Gelatin Hydrogel, WPCH: Whey Protein Concentrate Hydrogel).

**Table 3:** The texture parameters of the oleogels, hydrogels, and bigels.

Sample Name	Hardness (g)	Springness	Cohesiveness
Oleogel	626.55 ± 39.07 ^f^	0.976 ± 0.007	0.372 ± 0.060
CSGB-70%-O	3849.62 ± 93.75 ^b^	0.978 ± 0.002	0.992 ± 0.004
CSGB-50%-O	2928.56 ± 53.51 ^c^	0.985 ± 0.004	0.975 ± 0.005
CSGB-30%-O	749.78 ± 73.20 ^ef^	0.983 ± 0.000	1.004 ± 0.002
GB-70%-O	4300 ± 31.64 ^a^	0.995 ± 0.000	1.218 ± 0.040
GB-50%-O	3550.62 ± 17.91 ^b^	0.996 ± 0.001	0.826 ± 0.032
GB -30%-O	1758.36 ± 52.51 ^d^	0.993 ± 0.003	0.830 ± 0.016
WPC B-70%-O	3330.94 ± 242.13 ^b^	0.918 ± 0.035	0.929 ± 0.013
WPC B-50%-O	2517.07 ± 30.05 ^d^	0.977 ± 0.014	0.99 ± 0.001
WPC B-30%-O	645.87 ± 55.96 ^f^	0.982 ± 0.002	0.991 ± 0.007
CSGH	607.912 ± 63.11 ^f^	0.9835 ± 0.002	0.9812 ± 0.008
GH	659.41 ± 4.234 ^f^	0.997 ± 0.001	0.728 ± 0.040
WPCH	774.79 ± 41.81 ^e^	0.988 ± 0.004	0.590 ± 0.002

a–f Different superscript letters indicate significant differences between samples (*p* < 0.05). (*CSGB-70%-O, CSGB-50%-O, CSGB-30%-O: samples produced with cold pressed chia seed oil by-product gum at different oleogel concentrations, GB-70%-O, GB-50%-O, GB-30%-O: samples contained gelatin at different oleogel concentrations, WPCB-70%-O, WPCB-50%-O, WPCB-30%-O: samples contained Whey Protein Concentrate at different oleogel concentrations. CSGH: hydrogel produced from cold pressed chia seed oil by-product gum, GH: Gelatin Hydrogel, WPCH: Whey Protein Concentrate Hydrogel*).

**Table 4:** The rheological model parameters of the full-fat and low-fat mayonnaise produced from bigels.

Rheological Analysis	Parameters	FF	LF GB	LF CSGB	LF WPCB
Steady Shear	K (Pas^n^)	9.11 ± 0.65 ^b^	21.71 ± 1.43 ^a^	7.95 ± 0.57 ^b^	0.75 ± 0.01 ^c^
	n	0.32 ± 0.021	0.38 ± 0.019	0.29 ± 0.024	0.95 ± 0.078
	R^2^	0.999 ± 0.001	0.995 ± 0.002	0.99 ± 0.02	0.9950.003
Dynamic Rheological Behavior	*K*′ (Pas^n^)	11.31 ± 0.87 ^c^	145.60 ± 7.65 ^a^	19.48 ± 2.14 ^b^	0.166 ± 0.031 ^d^
	n′	0.55442 ± 0.04	0.1977 ± 0.001	0.32 ± 0.015	1.09 ± 0.05
	R^2^	0.978 ± 0.002	0.998 ± 0.001	0.987 ± 0.003	0.992 ± 0.004
	*K*″ (Pas^n^)	6.51 ± 0.33 ^b^	39.42 ± 3.15 ^a^	5.14 ± 0.24 ^b^	0.083 ± 0.004 ^c^
	n″	0.47 ± 0.001	0.21 ± 0.002	0.48 ± 0.003	1.102 ± 0.04
	R^2^	0.988 ± 0.001	0.995 ± 0.0014	0.997 ± 0.004	0.983 ± 0.003
3-ITT Rheological Behavior	Ge’	45.7± 3.27 ^b^	205 ± 11.24 ^a^	33.3 ± 1.54 ^b^	nm
	G_0′_	22.85 ± 1.89 ^b^	135 ± 6.53 ^a^	16.52 ± 0.87 ^b^	nm
	Ge’/Go’	2.00 ± 0.11 ^a^	1.51 ± 0.09 ^b^	1.97 ± 0.15 ^a^	nm
	k	5.2 ± 0.27 ^a^	4.52 ± 0.28 ^a^	4.65 ± 0.35 ^a^	nm
	R^2^	0.995 ± 0.02	0.915 ± 0.03	0.988 ± 0009	nm

a–d: Different superscript letters indicate significant differences between samples (*p* < 0.05). * nm: not modeled (*FF:Full Fat mayonnaise with 65% Oil, LF CSGB: Low-fat mayonnaise samples contained CSGB and 30% Oil, LF GB: Low-fat mayonnaise samples contained GB and 30% Oil, LF WPCB: Low-fat mayonnaise samples contained Whey Protein Concentrate Bigel and 30% Oil*).

## Data Availability

The data that support the findings of this study are available from the corresponding author upon reasonable request.
